# Feasibility of simultaneous development of laparoscopic and robotic pancreaticoduodenectomy

**DOI:** 10.1038/s41598-023-33269-x

**Published:** 2023-04-16

**Authors:** Ying-Jui Chao, Wei-Hsun Lu, Ting-Kai Liao, Ping-Jui Su, Chih-Jung Wang, Chao-Han Lai, Jo-Ying Hung, Pei-Fang Su, Yan-Shen Shan

**Affiliations:** 1 Department of Surgery, National Cheng Kung University Hospital, College of Medicine, Institute of Clinical Medicine, National Cheng Kung University, 138, Sheng-Li Road, Tainan, 70428 Taiwan; 2 Institute of Clinical Medicine, College of Medicine, National Cheng Kung University, 35 Siaodong Road, Tainan, 70457 Taiwan; 3 Department of Statistics, College of Management, National Cheng Kung University, No.1 University Road, Tainan, 70101 Taiwan

**Keywords:** Surgical oncology, Pancreatic disease

## Abstract

Laparoscopic (LPD) and robotic pancreaticoduodenectomy (RPD) are both challenging procedures. The feasibility and safety of simultaneously developing LPD and RPD remain unreported. We retrospectively reviewed the data of patients undergoing LPD or RPD between 2014 and 2021. A total of 114 patients underwent minimally invasive pancreaticoduodenectomy (MIPD): 39 LPDs and 75 RPDs. The learning process of LPD and RPD were similar. The cutoff points of the learning curve were LPD, 13th patient (the 27th patient of MIPD), and RPD, 18th patient (the 31st patient of MIPD) according the cumulative sum analysis of operative time. A decrease in the operative time was associated with the case sequence (p < 0.001) but not with the surgical approach (p = 0.36). The overall surgical outcomes were comparable between both the LPD and RPD groups. When evaluating the learning curve impact on MIPD, LPD had higher major complication (≧ Clavien–Dindo grade III), bile leak and wound infection rates in the pre-learning curve phase than those in the after-learning curve phase, while RPD had similar surgical outcomes between two phases. Simultaneous development of LPD and RPD is feasible and safe for experienced surgeons, with similar learning process and comparable surgical outcomes.

## Introduction

Pancreaticoduodenectomy (PD) is one of the most complex procedures of abdominal surgery that requires wide dissection and two delicate anastomoses. Although laparoscopic pancreaticoduodenectomy (LPD) and robotic pancreaticoduodenectomy (RPD) were first reported in 1994 and 2000, respectively^1,2^, the adoption and dissemination of minimally invasive pancreaticoduodenectomy (MIPD) were slow and unpopular owing to the steep learning curve. In LPD, surgeons should have sufficient experience with open pancreaticoduodenectomy (OPD) and advanced laparoscopic skills to be competent and shorten the learning curve. In experienced hands, 30–40 patients are required to overcome the learning curve of LPD^3–7^. Although the robotic system has benefits over laparoscopic surgery such as stable operative view, tremor filtration, and 7-degree mobility, the learning curve was similar to that of LPD and competed after 20–80 patients^8–10^. In general, the concepts and dissection planes of laparoscopic and robotic surgeries are similar, and both the approaches have the advantage of providing the caudal to cephalad view and using similar energy devices. Simultaneous development of laparoscopic and robotic surgery is feasible for major abdominal procedures. In gastric cancer surgery, the experience of robotic surgery can help overcome the learning curve of laparoscopic gastric cancer surgery^11^. Moreover, the experience of laparoscopic gastric cancer surgery can affect the learning curve of robotic gastric cancer surgery^12^.

Currently, the majority of surgeons only focus on either LPD or RPD, especially during the initial development period. Previous reports have compared the perioperative outcomes of LPD versus OPD or RPD versus OPD and have demonstrated that either LPD or RPD was potentially superior to OPD in terms of postoperative complications and hospital stay in well-selected patients^13–19^. The comparison of perioperative outcomes between LPD and RPD remains limited, and to the best of our knowledge, the feasibility of simultaneously developing LPD and RPD has not been reported in the literature to date. In addition, the learning curve effect should be considered when comparing the outcomes of these two approaches.

To address this gap in knowledge, this study aimed to investigate the feasibility of simultaneously developing LPD and RPD by comparing the learning curves and perioperative outcomes of LPD and RPD, determine the proficiency of LPD and RPD, and compare the outcomes of LPD and RPD at different learning curve phases.

## Materials and methods

### Institutional review board statement

All methods were carried out in accordance with the relevant guidelines and regulations. This study was approved by the Institutional Review Board of the National Cheng Kung University Hospital (Approval number: A-ER-110-441). Informed consent was waived by the Institutional Review Board of the National Cheng Kung University Hospital due to the anonymity and retrospective nature of this study.

### Data collection and outcome measurement

Data regarding clinicopathological characteristics and postoperative outcomes were collected retrospectively from December 2014 to March 2021. LPD has been performed since December 2014 at the National Cheng Kung University Hospital. Before the first LPD, we had performed 73 laparoscopic gastric cancer surgeries and 52 laparoscopic distal pancreatectomies. The da Vinci Si Surgical System (Intuitive Surgical Inc., Sunnyvale, CA, USA) was installed at our institution in December 2014. The first RPD was conducted in June 2015 after gaining experience with 10 robotic distal pancreatectomies and 8 robotic distal gastrectomies. All operations were performed by a single surgeon who was assisted by other experienced surgeons. Approximately 80 PDs were performed yearly at the National Cheng Kung University Hospital. Of the 552 patients who underwent PD between December 2014 and March 2021, 114 were MIPDs (LPD group, 39; RPD group, 75), whereas the remaining 437 patients underwent OPD. The potential advantages of RPD over LPD including the robotic endowrist with a 7 degree of freedom, ergonomic motion, and a stable surgical vision were well informed to the patients. However, the cost of RPD (3000 US dollars) is higher than that of LPD (15,000 US dollars). The final choice between RPD and LPD was decided by the patient’s preference. The operative time was defined as the time from skin incision to closure. Pancreatic texture was categorized as soft or hard by the surgeon’s intraoperative judgement via visual evaluation and instrument touching. The pancreatic duct width was measured from the preoperative computed tomography or magnetic resonance imaging. Dilated pancreatic duct was defined by the main pancreatic duct width > 3 mm.

Postoperative complications were recorded as any adverse event occurring within 90 days after surgery and stratified according to the Clavien–Dindo (CD) grade^20^. Postoperative pancreatic fistula (POPF), delayed gastric emptying (DGE), and postoperative pancreatectomy hemorrhage (PPH) were defined based on the International Study Group of Pancreatic Surgery^21–23^. Conversion was considered when MIPD was changed to open surgery. Reoperation was defined as a repeat operation due to severe complications after MIPD. Re-admission was recorded as any surgery-related admission within 30 days after discharge.

### Indications for LPD and RPD

The patients were carefully selected, and the indications for LPD and RPD were the same including periampullary tumors or gastric cancer with pancreatic head invasion and patients who could tolerate pneumoperitoneum. For pancreatic cancer, MIPD was indicated when the tumor size was < 2 cm and located at the pancreatic head due to concerns of tumor rupture and inadequate surgical margins. We excluded large tumors (> 10 cm), tumors with major vessel or other organ invasion, and tumors with bulky lymphadenopathy and patients with severe pancreatitis history and previous upper abdominal major surgery.

Another determining factor of MIPD was the cost of laparoscopic or robotic instruments, which was not covered by the Taiwan’s National Health Insurance and would affect the patients’ preference for OPD, LPD or RPD. The indications for MIPD did not expand after gaining experience or after the learning curve. Before the introduction of the da Vinci system, the laparoscopic approach was the only choice for MIPD, and eight patients underwent LPD during this period. The surgeon comprehensively explained the advantages and disadvantages of LPD, RPD, and OPD to the patients, and the decision was made by each patient.

### Surgical procedure

#### Robotic surgery

After induction of general anesthesia, a 30° reverse Trendelenburg position with leg-split was used, and the table was rotated 10° to the left. Pneumoperitoneum was established from the subumbilical port, and laparoscopic examination was performed to assess unexpected tumor peritoneal seeding or liver metastasis. The robotic system was docked to the patient’s head. Six trocars were used, and the assistant surgeon stood between the legs of the patient. The proximal jejunum was transected using a 45-mm white cartridge endostapler 20 cm distal to the ligament of Treitz. Extended Kocher’s maneuver was performed, and the proximal duodenum (or antrum) was divided by a 45-mm white or blue cartridge endostapler. The pylorus and antrum were sacrificed if tumor invasion or obvious duodenal ulcers were observed. The group 8a and 12 stationed lymph nodes were harvested, and the gastroduodenal artery was clipped and divided using hem-o-lock clips. Cholecystectomy and transection of the common bile duct were performed. The pancreatic neck was transected using ultrasonic shears. The uncinate process was dissected from the right margin of the superior mesenteric artery. The pancreaticojejunostomy (PJ) was completed using the modified Blumgart method, as described in our previous study^24^. An internal stent was placed if the pancreatic duct was < 5 mm in diameter. The hepaticojejunostomy (HJ) was performed using a one-layer continuous suturing with 4–0 or 5–0 prolene suture. For pylorus-preserving pancreaticoduodenectomy (PPPD), duodenojejunostomy was performed by mini-laparotomy after specimen retrieval. For a classical PD, the gastrojejunostomy was completed intracorporeally.

#### Laparoscopic surgery

The surgical technique for LPD has been previously published^24^. After induction of general anesthesia, the patient was placed in a 30° reverse Trendelenburg position with the leg-split. Pneumoperitoneum was established through the umbilical port, and further procedures were performed after excluding unexpected peritoneal seeding or liver metastasis. At the resection stage, the surgeon stood on the left side of the patient, the camera surgeon between the patient’s legs, and the assistant surgeon on the right side of the patient. The proximal jejunum was transected, followed by transection of the 1st part of the duodenum or antrum, group 8a and 12 lymph node dissection, gastroduodenal artery transection, bile duct transection, and cholecystectomy. The surgeon then moved to the right of the patient and the assistant surgeon to the left. Extended Kocher’s maneuver was performed, followed by pancreatic neck transection and uncinate process dissection. During the reconstruction stage, the surgeon stood between the legs of the patient, and a modified Blumgart PJ was performed with an internal stent. The surgeon then returned to the right of the patient to complete the HJ and gastrojejunostomy/duodenojejunostomy. Generally, the resection and reconstruction procedures and methods of LPD are similar to those of RPD.

### Statistical analysis

Data analysis included descriptive and inferential statistics. Descriptive statistics are presented as the estimated median (interquartile range) for continuous variables and frequencies for categorical variables. To examine categorical variables, the chi-squared test or Fisher’s exact test was used. The Mann–Whitney U test was used to examine continuous variables. To identify whether the dependent variables (e.g., RPD, LPD, and patient sequence) were associated with the outcome or operative time, multiple linear regression models were used. The learning curves of RPD and LPD were determined using cumulative sum (CUSUM) analysis and defined as ∑ (Xi − X0), where X0 is the mean value of the operative time, and Xi is the operative time of each patient. All statistical tests were two-sided, and p < 0.05 was considered significant. Statistical analyses were performed using the Statistical Product and Service Solutions (SPSS) (version 19.0; IBM SPSS, Chicago, IL, USA) and R software, version 4.0.2 (R Foundation for Statistical Computing, Vienna, Austria).

## Results

### Comparison of perioperative results between LPD and RPD

During the study period, 114 consecutive patients underwent MIPD: 39 LPDs and 75 RPDs (Table 1). The median age and body mass index (BMI) of the patients were 66.1 years and 23.8 kg/m^2^, respectively. The pathological diagnoses were ampullary cancer, 35.1%; pancreatic cancer, 14.9%; cholangiocarcinoma, 13.2%; duodenal cancer, 0.9%; other malignancies, 5.3%; and benign disease, 30.7%. The median operative time and blood loss were 302 min and 100 mL, respectively (Table 2). PPPD was performed in 84.2% of patients. Five patients (4.4%) were converted to open surgery. Three patients (7.7%) required conversion to open surgery in the LPD group because of unexpected superior mesenteric vein invasion (n = 1) and severe inflammation around the superior mesenteric vein (n = 2), and two patients (2.7%) required to be converted to open surgery in the RPD group because of severe inflammation around the superior mesenteric vein. The overall major complication rate (CD ≥ III) in MIPD was 20.2%. There were 18.4% CR-POPF, 11.4% DGE, 11.4% PPH, 7% bile leak, 16.5% postoperative fluid collection, 12.3% wound infection, and 1.8% reoperation rates. The median length of hospital stay was 15 days, with an 8.8% re-admission rate. One patient (1.3%) died in the RPD group secondary to PPH and grade C POPF.

**Table 1:** Patient demographics of MIPD.

	MIPD	LPD	RPD	p value
Patients, n	114	39	75	
Sex, M/F	57/57	15/24	42/33	0.08
Age, years	66.1 (58.2–75.2)	67.1 (58.3–74.6)	65.5 (58.1–75.5)	0.53
BMI, kg/m^2^	23.8 (22.2–25.9)	23.7 (21.2–25.6)	23.8 (22.3–27)	0.32
ASA score				0.17
I	6 (5.3%)	0	6 (8%)	
II	63 (55.3%)	24 (61.5%)	39 (52%)	
III	45 (39.5%)	15 (38.5%)	30 (40%)	
Diagnosis				0.81
Ampullary cancer	40 (35.1%)	15 (38.5%)	25 (33.3%)	
Pancreatic cancer	17 (14.9%)	4 (10.3%)	13 (17.3%)	
Cholangiocarcinoma	15 (13.2%)	5 (12.8%)	10 (13.3%)	
Duodenal cancer	1 (0.9%)	0	1 (1.3%)	
Other malignancy	6 (5.3%)	3 (7.7%)	3 (4%)	
Benign disease	35 (30.7%)	12 (30.8%)	23 (30.7%)	
Tumor size, cm	2 (1.2–3)	2 (1.2–3.5)	2 (1–3)	0.41
Soft pancreas	106 (93%)	36 (92.3%)	71 (94.7%)	0.69
Dilated pancreatic duct	33 (28.9%)	15 (38.5%)	18 (24%)	0.13
Learning curve phase				0.38
PLC phase		13 (33.3%)	18 (24%)	
ALC phase		26 (66.7%)	57 (76%)	

*BMI* body mass index, *ASA* American Society of Anesthesiology, *PLC* pre-learning curve, *ALC* after-learning curve.

**Table 2:** Perioperative outcomes of MIPD.

	MIPD	LPD	RPD	p value
Patients, n	114	39	75	
Operative time, min	302 (266–361)	307 (274–370)	298 (263–360)	0.45
Blood loss, mL	100 (0–200)	100 (0–300)	100 (0–200)	0.89
Procedure				0.79
PPPD	96 (84.2%)	32 (82.1%)	64 (85.3%)	
Whipple	18 (15.8%)	7 (17.9%)	11 (14.7%)	
Harvested lymph node, n	15.5 ± 9.7	17.3 ± 13.3	14.6 ± 7.2	0.25
Open conversion	5 (4.4%)	3 (7.7%)	2	0.34
CD grade				0.70
0	55 (48.2%)	16 (41%)	39 (52%)	
I	10 (8.8%)	5 (12.8%)	5 (6.7%)	
II	27 (23.7%)	10 (25.6%)	17 (22.7%)	
IIIa	13 (11.4%)	4 (10.3%)	9 (12%)	
IIIb	1 (0.9%)	1 (2.6%)	0	
IVa	5 (4.4%)	2 (5.1%)	3 (4%)	
IVb	2 (1.8%)	1 (2.6%)	1 (1.3%)	
V	1 (0.9%)	0	1 (1.3%)	
Major complication, CD ≧ III	23 (20.2%)	8 (20.5%)	15 (20%)	> 0.99
DGE, grade B + C	13 (11.4%)	4 (10.3%)	9 (12%)	> 0.99
PPH, grade B + C	13 (11.4%)	4 (10.3%)	8 (10.7%)	> 0.99
POPF				0.35
Biochemical	43 (37.7%)	11 (28.2%)	32 (42.7%)	
B	20 (17.5%)	9 (23.1%)	11 (14.7%)	
C	1 (0.9%)	0	1 (1.3%)	
Bile leak	8 (7%)	3 (7.7%)	5 (6.7%)	> 0.99
Fluid collection	19 (16.7%)	6 (15.4%)	13 (17.3%)	> 0.99
Wound infection	14 (12.3%)	5 (12.8%)	9 (12%)	> 0.99
Reoperation	2 (1.8%)	1 (2.6%)	1 (1.3%)	> 0.99
Hospital stay, days	15 (12–23)	15 (12–23)	15 (11–23)	0.31
Re-admission	10 (8.8%)	1 (2.6%)	6 (8%)	0.67

*PPPD* pylorus preserving pancreaticoduodenectomy, *CD* Clavien–Dindo, *DGE* delayed gastric emptying, *PPH* postpancreatectomy hemorrhage, *POPF* postoperative pancreatic fistula.

Comparing the LPD and RPD groups, the preoperative demographic data, including sex, age, BMI, American Society of Anesthesiology (ASA) score, pathological diagnosis, tumor size, pancreatic gland texture, the ratio of dilated pancreatic duct, and the distribution of learning curve phase showed no significant difference (all p > 0.05; Table 1). The operative time, blood loss, pylorus-preserving rate, and harvested lymph nodes were similar between the two groups (Table 2). The surgical outcomes, including overall complications, major complications, CR-POPF, DGE, PPH, bile leak, postoperative fluid collection, wound infection, reoperation, length of hospital stay, and re-admission were comparable between the groups.

### Learning process of LPD, RPD, and MIPD

**Figure 1:**
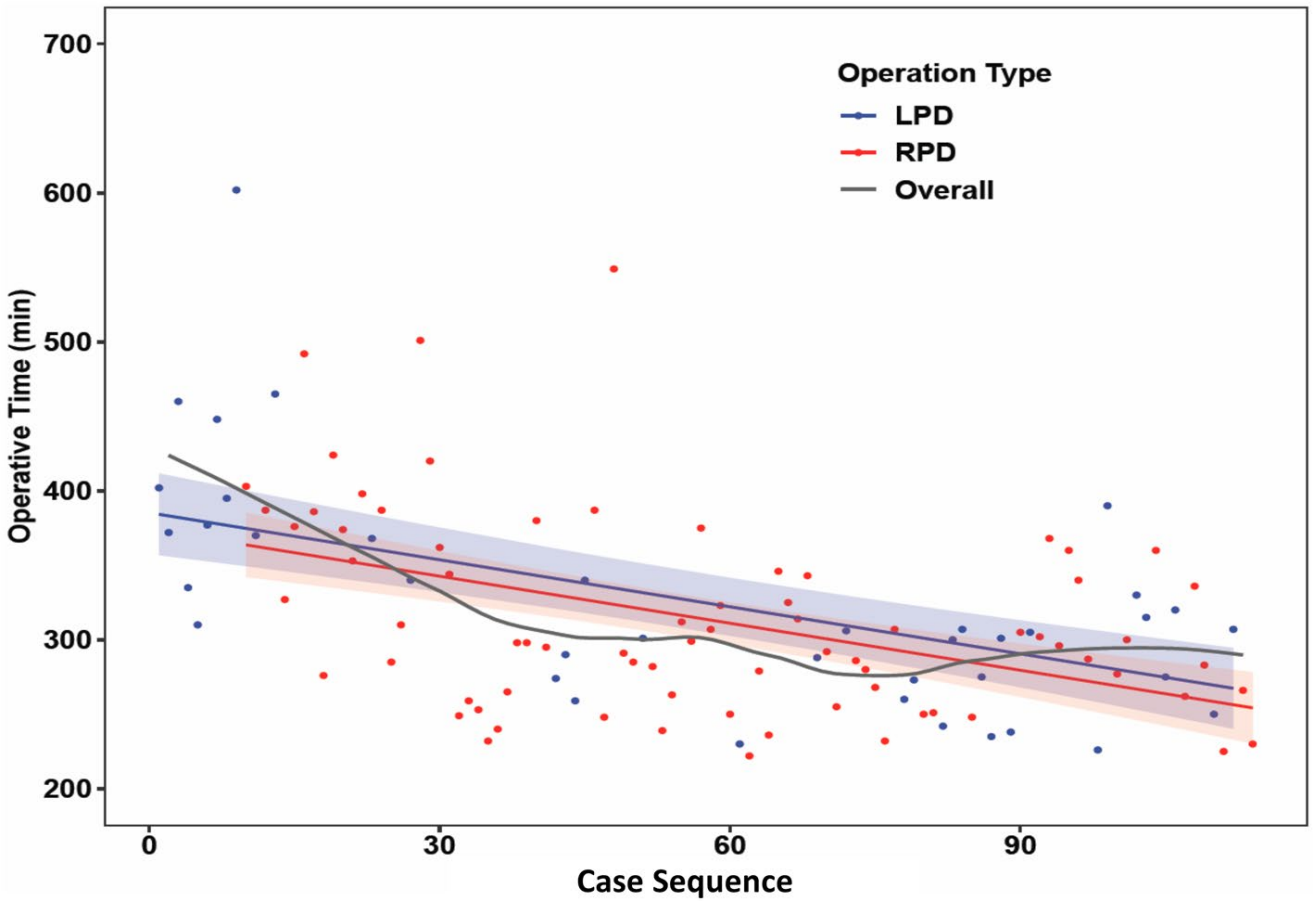
The operative time versus patient sequence of MIPD in a scatter plot. The black line showed the moving average of the operative time. The blue and red solid line presented as the regression line of operative time and the operative time was decreasing for both LPD (blue) and RPD (red).

**Figure 2:**
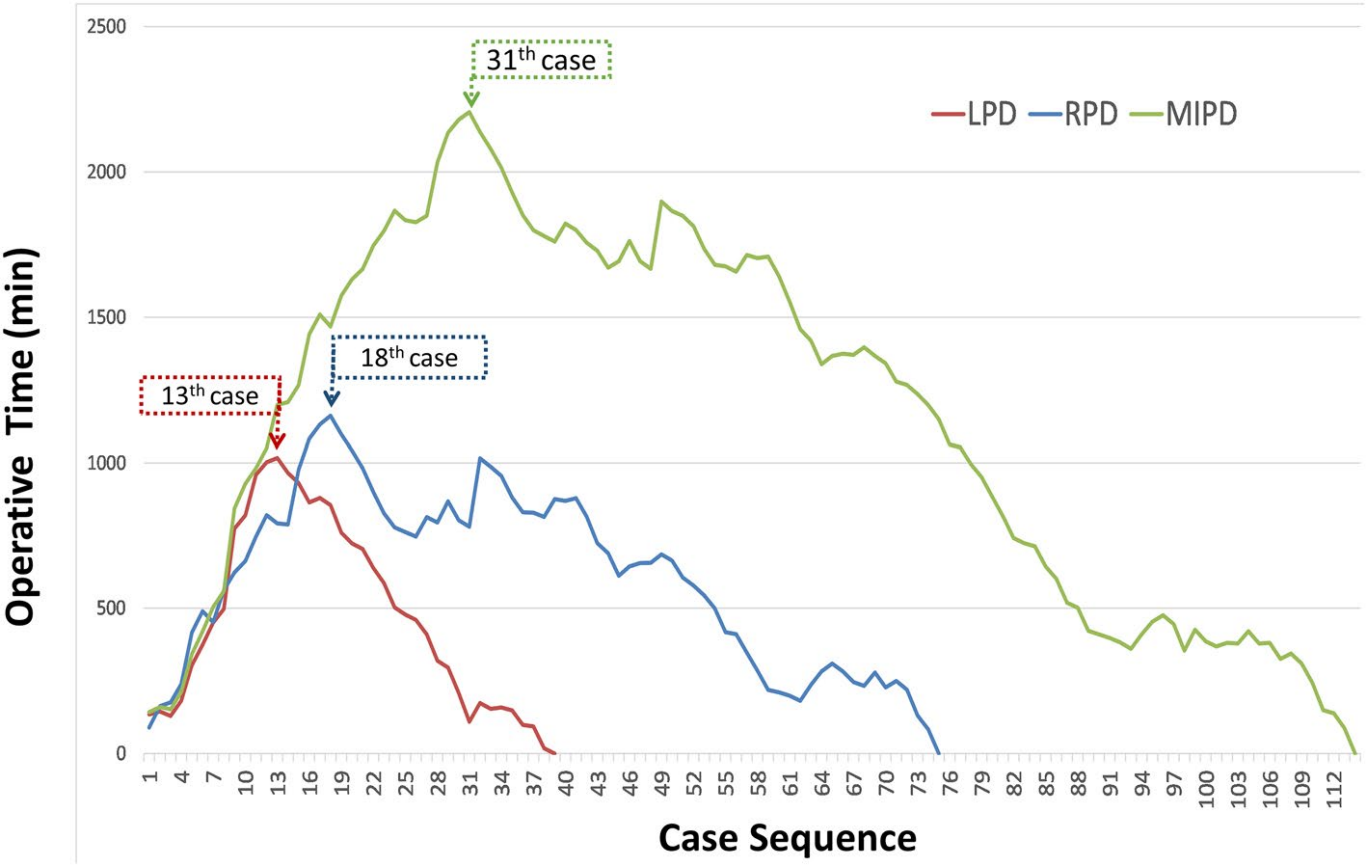
Cumulative sum analysis of the operative time. An uphill slope and down slope indicate an increasing trend and decreasing trend, respectively. The peak indicates the point of overcoming the learning curve. Overall, 13, 18, and 31 patients are required to be proficient in LPD, RPD, and MIPD, respectively.

The operative times of LPD and RPD were plotted together for each case in chronological order. The first RPD was performed after gaining experience with eight LPDs (Fig. 1). The moving average of the operative time for all 114 patients showed a decreasing pattern. The operative time was correlated with the case sequence of MIPD, whereas both the LPD and RPD groups demonstrated decreasing trends. In the multiple regression model, the decline in operative time was strongly associated with the case sequence (1.05 min decrease per case, p < 0.001) but not with the operation type (p = 0.36; Table 3). Further, we evaluated the learning curve using CUSUM analysis of the operative time (Fig. 2). The peaks, as the cutoff points of the learning curve for LPD and RPD, were observed in the 13th case of LPD (the 27th of MIPD) and the 18th case of RPD (the 31st of MIPD) (Fig. 2). Considering the proficiency of MIPD, 31 cases were required to overcome the learning curve. The time points of passing the learning curve were close among LPD, RPD, and MIPD, indicating that the case number affected the learning curve of MIPD more than the operation type.

**Table 3:** Multivariable regression for operation time in operation type and case sequence.

Variables		Coefficient (SE)	p value
Intercept		385.29 (13.98)	< 0.001
Operation type	LPD	Ref	
	RPD	− 11.03 (12.09)	0.36
Case sequence		− 1.05 (0.17)	< 0.001

### Perioperative results on the learning curve phases: pre-learning curve (PLC) phase vs after-learning curve (ALC) phase in LPD and RPD groups

In LPD, a male predominance (100% vs. 42%, p < 0.001) and younger age (median, 58 vs. 72 years old; p = 0.02) were observed in the PLC group than in the after-learning curve (ALC) group (Supplementary Table 1). Other preoperative characteristics were comparable between the two phases. The operative time was significantly longer in the ALC phase than in the PLC phase (median, 377 min vs. 289 min; p < 0.001). Although the overall complication was not significantly different between the two phases, the major complication rate (46% vs. 8%, p = 0.01), bile leak rate (23% vs. 0%, p = 0.03), and wound infection rate (38% vs. 8%, p = 0.03) were significantly higher in the PLC phase than in the ALC phase (Table 4). Among patients with bile leak in the PLC phase, two patients were associated with POPF and one patient experienced a pure/isolated bile leak. Meanwhile, CR-POPF, DGE, postoperative fluid collection, reoperation, length of hospital stay, and re-admission rates were similar between the two phases.

**Table 4:** Perioperative outcomes between learning curve phases in LPD and RPD.

	LPD			RPD		
	PCL phase	ALC phase	p value	PCL phase	ALC phase	p value
Patients, n	13	26		18	57	
PPPD/whipple	10/3	22/4	0.67	15/3	49/8	0.72
Operative time, min	377 (354–454)	289 (257–307)	< 0.001	381 (340–407)	286 (252–313)	< 0.001
Blood loss, mL	200 (0–350)	50 (0–200)	0.12	100 (100–100)	100 (0–100)	0.14
Open conversion	1 (8%)	2 (8%)	> 0.99	0	2 (4%)	> 0.99
Harvested lymph nodes, n	17 (7.5–22.5)	14.5 (9–21.3)	0.73	14.5 (8–21)	13 (10–19)	0.96
CD grade			0.11			0.85
0	4 (31%)	12 (46%)		10 (56%)	29 (51%)	
I	2 (15%)	3 (12%)		0	5 (9%)	
II	1 (8%)	9 (35%)		5 (28%)	12 (21%)	
IIIa	3 (23%)	1 (4%)		2 (11%)	7 (12%)	
IIIb	1 (8%)	0		0	0	
IVa	1 (8%)	1 (4%)		1 (6%)	2 (4%)	
IVb	1 (8%)	0		0	1 (2%)	
V	0	0		0	1 (2%)	
Major complication, CD ≧ III	6 (46%)	2 (8%)	0.01	3 (17%)	12 (21%)	> 0.99
POPF			0.41			0.21
Biochemical	2 (15%)	9 (35%)		8 (44%)	24 (42%)	
B	3 (23%)	6 (23%)		1 (6%)	10 (18%)	
C	0	0		1 (6%)	0	
DGE, grade B + C	3 (23%)	1 (4%)	0.10	2 (11%)	7 (12%)	> 0.99
PPH, grade B + C	2 (15%)	2 (8%)	0.59	3 (17%)	6 (11%)	0.44
Bile leak	3 (23%)	0	0.03	0	5 (9%)	0.33
Fluid collection	4 (31%)	2 (8%)	0.15	2 (11%)	11 (19%)	0.72
Wound infection	5 (38%)	2 (8%)	0.03	1 (6%)	8 (14%)	0.68
Reoperation	1 (8%)	0	0.33	0	1 (2%)	> 0.99
Hospital stay, days	17 (12.5–30)	15 (12–20)	0.26	13 (11–16.3)	15 (11–23)	0.53
Re-admission	2 (15%)	0	0.11	3 (17%)	5 (9%)	0.39

*PLC* pre-learning curve, *ALC* after-learning curve, *PPPD* pylorus preserving pancreaticoduodenectomy, *CD* Clavien–Dindo, *DGE* delayed gastric emptying, *PPH* postpancreatectomy hemorrhage, *POPF* postoperative pancreatic fistula.

In RPD, the preoperative clinical characteristics showed no difference between the PLC and ALC phases (Supplementary Table 1). The operative time was significantly shorter in the ALC phase than in the PLC phase (median, 381 vs. 286 min; p < 0.001). The overall complications, major complications, CR-POPF, DGE, PPH, bile leak, postoperative fluid collection, reoperation, length of hospital stay, and re-admission rates were comparable between the phases (Table 5).

**Table 5:** Perioperative outcomes between approaches in the PLC and ALC phase.

	PLC phase			ALC phase		
	LPD	RPD	*p* value	LPD	RPD	*p* value
Patients, n	13	18		26	57	
PPPD/whipple	10/3	15/3	0.68	22/4	49/8	> 0.99
Operative time, min	377 (354–454)	381 (340–407)	0.56	289 (257–307)	286 (252–313)	0.91
Blood loss, mL	200 (0–350)	100 (100–100)	0.85	50 (0–200)	100 (0–100)	0.63
Open conversion	1 (8%)	0	0.42	2 (8%)	2 (4%)	0.59
Harvested lymph nodes, n	17 (7.5–22.5)	14.5 (8–21)	0.62	14.5 (9–21.3)	13 (10–19)	0.69
CD grade			0.18			0.63
0	4 (31%)	10 (56%)		12 (46%)	29 (51%)	
I	2 (15%)	0		3 (12%)	5 (9%)	
II	1 (8%)	5 (28%)		9 (35%)	12 (21%)	
IIIa	3 (23%)	2 (11%)		1 (4%)	7 (12%)	
IIIb	1 (8%)	0		0	0	
IVa	1 (8%)	1 (6%)		1 (4%)	2 (4%)	
IVb	1 (8%)	0		0	1 (2%)	
V	0	0		0	1 (2%)	
Major complication, CD ≧ III	6 (46%)	3 (17%)	0.11	2 (8%)	12 (21%)	0.53
POPF			0.18			0.44
Biochemical	2 (15%)	8 (44%)		9 (35%)	24 (42%)	
B	3 (23%)	1 (6%)		6 (23%)	10 (18%)	
C	0	1 (6%)		0	0	
DGE, grade B + C	3 (23%)	2 (11%)	0.63	1 (4%)	7 (12%)	0.71
PPH, grade B + C	2 (15%)	3 (17%)	> 0.99	2 (8%)	6 (11%)	> 0.99
Bile leak	3 (23%)	0	0.06	0	5 (9%)	0.17
Fluid collection	4 (31%)	2 (11%)	0.21	2 (8%)	11 (19%)	0.21
Wound infection	5 (38%)	1 (6%)	0.06	2 (8%)	8 (14%)	0.49
Reoperation	1 (8%)	0	0.42	0	1 (2%)	> 0.99
Hospital stay, days	17 (12.5–30)	13 (11–16.3)	0.09	15 (12–20)	15 (11–23)	0.90
Re-admission	2 (15%)	3 (17%)	> 0.99	0	5 (9%)	0.17

*PLC* pre-learning curve, *ALC* after-learning curve, *PPPD* pylorus preserving pancreaticoduodenectomy, *CD* Clavien–Dindo, *DGE* delayed gastric emptying, *PPH* postpancreatectomy hemorrhage, *POPF* postoperative pancreatic fistula.

### Comparison of perioperative results in the phases between groups: LPD vs RPD in the PLC and ALC phase

In the PLC phase, the LPD group had a male predominance (p < 0.001), lower ASA grade (p = 0.06), and more patients with ampullary cancer (p = 0.06) than in the RPD group (Supplementary Table 2). The operative time, blood loss, pylorus-preserving rate, harvested lymph nodes, overall complications, major complications, POPF, DGE, PPH, reoperation, and length of hospital stay were comparable between LPD and RPD in the PLC phase, except for a marginally higher risk of bile leak (23% vs. 0%, p = 0.06), wound infection rate (38% vs. 6%, p = 0.06), and longer hospital stay (median, 17 vs. 13 days, p = 0.091) in the LPD group (Table 5). In the ALC phase, no significant difference was noted in the preoperative characteristics between LPD and RPD, except for slightly older patients in the LPD group (median, 72 vs 65.5 years old, p = 0.07). Postoperative morbidities were similar between the two groups.

## Discussion

With the development of robotic systems, robotic platforms provide another approach to minimally invasive surgery, and the simultaneous development of laparoscopic and robotic abdominal surgery has become an emerging model^25^. Moreover, previous experience with either robotic or laparoscopic surgery affects the learning process to the other approach^11,12^. In this study, we demonstrated that simultaneously developing LDP and RPD was safe and feasible for experienced surgeons during the initial learning period of MIPD. The surgical outcomes of LPD and RPD were comparable in terms of PODF, DGE, PPH, bile leak, fluid collection, wound infection, reoperation, and length of hospital stay. Otherwise, the improvement of operative time mainly depended on the patient numbers of MIPD performed but not associated with the operation type, and the operative time decreased by 1.05 min per patient in sequence during this period. In addition, LPD and RPD had comparable operative times and a similar declining trend of operative time as the experience of MIPD is gained. In general, the dissection and operative views were similar between these two approaches, and the experience of the laparoscopic or robotic approach can decrease the operative time in either of the procedures. Hence, the time points of the learning curve were close between LPD and RPD (LPD, 27th patient of MIPD; RPD, 31st patient of MIPD).

The superiority of the robotic or laparoscopic approach in MIPD remains debatable, and the comparison of LPD and RPD should be considered under the proficiency and learning curve effect. To the best of our knowledge, the present study is the first report to show the surgical results of LPD and RPD at different phases of the learning curve in a simultaneous development model. Currently, only a few studies have directly compared the results of LPD and RPD^25–27^. A randomized control trial for LPD and RPD is difficult to conduct because of the high cost of the robotic platform, variation of disease type, and surgeon experience. Liu et al. reported the first head-to-head study to compare the surgical outcomes of LPD and RPD in the PLC phase. They concluded that RPD had a shorter operative time, lower estimated blood loss, and shorter hospital stay than LPD, although the overall postoperative complications were comparable between the two approaches^25^. Recently, Oosten et al. used the propensity score matching (PSM) method to eliminate the bias of patient selection between LPD and RPD^26^. However, the surgery year was still a bias after PSM use because LPD was developed earlier than RPD as the dates have limited overlap between these two approaches. The impact of proficiency would exist, and the RPD group may have the advantage of learning experience from the LPD group. Eventually, they identified that RPD had a longer operative time, less blood loss, and higher blood transfusion rate than LPD; otherwise, both approaches had similar postoperative complications. Vining et al. reported the largest multicenter study (LPD, 407 patients; RPD, 498 patients) from the American College of Surgeons National Surgical Quality Improvement Program database. LPD had a higher rate of vascular resection (16.7% vs. 9.0%, p = 0.005), concomitant visceral resection (4.4% vs. 0.8%, p = 0.004), blood transfusion (17.4% vs. 10.4%, p = 0.02), and conversion to open surgery (40.8% vs. 13.5%, p < 0.001) than RPD, which may reflect the selection bias of MIPD, and the severity of disease in LPD was higher than in RPD^28^. Subsequently, LPD led to a longer duration to drain removal (7 vs. 4 days, p < 0.001), more percutaneous drain placement (15.7% vs. 10.8%, p = 0.03), and longer hospital stay. However, the re-admission rate of RPD was higher than that of LPD (24.3% vs. 15.5%, p = 0.001). A network meta-analysis showed no significant difference between RPD and LPD for major complications, pancreatic specific complications, bile leak, mortality, and R0 resections^27^. However, RPD had a longer operative time, lower transfusion rates, and lower conversion rates than LPD.

In the present study, the benefits of RPD over LPD in the PLC phase were slightly lower major complications, fewer bile leaks, lower wound infections, and shorter length of hospital stay. The advantages of RPD over LPD attribute to the endowrist, ergonomic motion, tremor elimination, and a stable surgical vision of the robotic platform, which would improve the surgical dexterity and outcomes of PJ and HJ. In contrast, LPD requires more designed/standardized procedures and advanced suture techniques for pancreatic and biliary reconstruction. For example, we need to perform the duct-to-mucosa anastomosis of PJ via different working ports in LPD. Besides, a longer operative time will increase hand tremor in LPD, which may decrease the fine movement of surgery and increase the failure rate of PJ or HJ. Hence, robotic approach for PD provides a more accessible platform and simplifies the procedures for PJ and HJ compared to laparoscopic approach, which may reduce the surgical challenges and complications during the initial learning curve phase. However, when the learning curve was overcome and the surgical procedure was standardized, the advantages of RPD diminished, and the surgical outcomes were comparable between the two groups.

When considering the impact of proficiency on LPD, higher major complication rates, bile leak rate, wound infection rates, and slightly higher DGE rates were observed in the PLC phase than in the ALC phase. Although a higher bile leak rate was observed in the PLC phase of LPD, two patients with bile leaks were associated with POPF and experienced major complications. POPF remained the most critical complication in PD and might lead to severe complications. Kim et al. described that the surgical outcomes improved after overcoming the learning curve of LPD, and the complication rate decreased from 33 to 17.6%^7^. Wang et al. reported that complication rates (CD ≥ III) increased from 9.1% and 7.4% to 21.1%, respectively, through the learning curve phases associated with an increased percentage of LPD from 35.5%, 54%, and 76%. A higher complication rate after the learning curve may be attributed to expanding the inclusion criteria and involving more challenging cases after gaining experience^5^. Hence, expanding the criteria during the implementation of MIPD should be weighed to evaluate the outcomes during the two phases. In the present study, the inclusion criteria were consistent between the two phases, and the ratio of MIPDs/PDs in our study was similar between the peri-learning curve phases (PLC phase, 21.5%; ALC phase, 25.9%). Hence, the selection bias of expanding criteria between the phases of MIPD was minimized in this study.

In contrast, in RPD, previous studies showed similar results regarding the impact of proficiency on surgical outcomes. Napoli et al. reported that the major complication rates (≥ Clavien–Dindo grade III) were comparable between the peri-learning curve phases of RPD, except for a higher DGE rate in the PLC curve^8^. Zhang et al. described that there was no difference in major complications (≥ Clavien–Dindo grade III) between the two phases; however, increased DGE, PPH, and reoperation and longer hospital stay were noted in the PLC phase of RPD^29^. Later, Shyr et al. identified that the complication rates were not different between the two phases^30^. In our study, the RPD had equivalent complication rates between the peri-learning curve phases. Hence, the robotic approach for PD may have the advantage of not increasing the surgical risks when compared with the laparoscopic approach.

This study had several limitations. First, this was a retrospective study, and selection bias would inevitably exist. Although LPD developed earlier than RPD, the majority of patients underwent RPD. The number of LPD less than RPD was due to that the potential advantages of RPD were well informed to the patients, including the robotic endowrist with a 7 degree of freedom, ergonomic motion, tremor elimination, and a stable surgical vision, which might improve the surgical dexterity and outcomes of PJ and HJ. The reasons for patients not selecting RPD were mainly due to the high cost and unavailability of the da Vinci system. In the PLC phase of LPD, the initial four patients were carefully selected (dilated bile duct and pancreatic duct); hence, more patients with dilated pancreatic duct were noted in the PLC phase as compared to the ALC phase (46% vs. 22%, p = 0.25). Subsequently, the inclusion criteria for LPD and RPD were equal and consistent. The risk factors of POPF, including age, BMI, pancreatic gland texture, disease type, and pancreatic duct diameter, were not contraindications for LPD. Second, the case number of LPD was relatively small, which might affect the drawing of learning phase. Despite the small case number of LPD, the time points of passing the learning curve were close LPD, RPD, and MIPD (LPD, 27th patient of MIPD; RPD, 31st patient of MIPD; MIPD, 31st patient of MIPD) as the implementation of an MIPD program. The learning curves of RPD and LPD tended to decrease with adherence to a well-established procedure of MIPD. The decline of operative time of MIPD mainly attributed to case number but not the operation type (LPD or RPD). However, a large patient cohort study is warranted to verify the results. Third, we had a low percentage of pancreatic cancer patients because of the strict indication for pancreatic cancer and the concerns of tumor rupture and inadequate surgical margins during the implantation period of MIPD. However, there was no significant difference in the disease types between LPD and RPD. In the current era of minimally invasive surgery, laparoscopic and robotic approach are feasible to various abdominal surgeries. This study provided a new insight of feasibility of simultaneous development for LPD and RPD and identified worse outcomes in the PLC phase than in the ALC phase in LPD. RPD may play a protective role before achieving proficiency in MIPD, and further studies are required to clarify the proficiency effect of the different approaches.

In conclusion, simultaneously developing LPD and RPD as an implementation of MIPD is safe and feasible for experienced surgeons. The surgical outcomes of LPD and RPD were comparable. When evaluating the learning impact on MIPD, LPD may have higher complication rates in the PLC than in the ALC phase, and the robotic approach may have the advantage of not increasing the surgical risks in the PLC phase. RPD seems to diminish the effect of proficiency on complications. Surgeons should evaluate potential benefits and surgical risks of different approach in different phases to construct a strategy for successful implementation of MIPD. Further studies are warranted to validate these results.

## Supplementary Information

Supplementary Tables.

## Data Availability

The datasets used in this study are available from the corresponding author upon reasonable request and are not available in public.

## References

[1] Gagner M, Pomp A (1994). Laparoscopic pylorus-preserving pancreatoduodenectomy. Surg. Endosc..

[2] Giulianotti PC (2010). Robot-assisted laparoscopic pancreatic surgery: Single-surgeon experience. Surg. Endosc..

[3] Nagakawa Y (2018). Learning curve and surgical factors influencing the surgical outcomes during the initial experience with laparoscopic pancreaticoduodenectomy. J. Hepatobiliary Pancreat. Sci..

[4] Lu C (2016). Analysis of learning curve for laparoscopic pancreaticoduodenectomy. J. Vis. Surg..

[5] Wang M (2016). Learning curve for laparoscopic pancreaticoduodenectomy: A CUSUM analysis. J. Gastrointest. Surg..

[6] Speicher PJ (2014). Defining the learning curve for team-based laparoscopic pancreaticoduodenectomy. Ann. Surg. Oncol..

[7] Kim SC (2013). Short-term clinical outcomes for 100 consecutive cases of laparoscopic pylorus-preserving pancreatoduodenectomy: Improvement with surgical experience. Surg. Endosc..

[8] Napoli N (2016). The learning curve in robotic pancreaticoduodenectomy. Dig. Surg..

[9] Boone BA (2015). Assessment of quality outcomes for robotic pancreaticoduodenectomy: Identification of the learning curve. JAMA Surg..

[10] Zhang T, Zhao ZM, Gao YX, Lau WY, Liu R (2018). The learning curve for a surgeon in robot-assisted laparoscopic pancreaticoduodenectomy: A retrospective study in a high-volume pancreatic center. Surg. Endosc..

[11] Huang KH (2014). Comparison of the operative outcomes and learning curves between laparoscopic and robotic gastrectomy for gastric cancer. PLoS ONE.

[12] Kim HI, Park MS, Song KJ, Woo Y, Hyung WJ (2014). Rapid and safe learning of robotic gastrectomy for gastric cancer: Multidimensional analysis in a comparison with laparoscopic gastrectomy. Eur. J. Surg. Oncol..

[13] Zimmerman AM, Roye DG, Charpentier KP (2018). A comparison of outcomes between open, laparoscopic and robotic pancreaticoduodenectomy. HPB (Oxford).

[14] Pedziwiatr M (2017). Minimally invasive versus open pancreatoduodenectomy-systematic review and meta-analysis. Langenbecks Arch. Surg..

[15] Shin SH (2017). Totally laparoscopic or robot-assisted pancreaticoduodenectomy versus open surgery for periampullary neoplasms: Separate systematic reviews and meta-analyses. Surg. Endosc..

[16] de Rooij T (2016). Minimally invasive versus open pancreatoduodenectomy: Systematic review and meta-analysis of comparative cohort and registry studies. Ann. Surg..

[17] Peng L, Lin S, Li Y, Xiao W (2017). Systematic review and meta-analysis of robotic versus open pancreaticoduodenectomy. Surg. Endosc..

[18] Zureikat AH (2016). A multi-institutional comparison of perioperative outcomes of robotic and open pancreaticoduodenectomy. Ann. Surg..

[19] Palanivelu C (2017). Randomized clinical trial of laparoscopic versus open pancreatoduodenectomy for periampullary tumours. Br. J. Surg..

[20] Dindo D, Demartines N, Clavien PA (2004). Classification of surgical complications: A new proposal with evaluation in a cohort of 6336 patients and results of a survey. Ann. Surg..

[21] Wente MN (2007). Delayed gastric emptying (DGE) after pancreatic surgery: A suggested definition by the International Study Group of Pancreatic Surgery (ISGPS). Surgery.

[22] Bassi C (2017). The 2016 update of the International Study Group (ISGPS) definition and grading of postoperative pancreatic fistula: 11 Years After. Surgery.

[23] Wente MN (2007). Postpancreatectomy hemorrhage (PPH): An International Study Group of Pancreatic Surgery (ISGPS) definition. Surgery.

[24] Chao, Y.-J. & Shan, Y.-S. In *Innovation of Diagnosis and Treatment for Pancreatic Cancer* (ed. Hiroki, Y.) 129–145 (Springer Singapore, 2017).

[25] Liu R (2017). The surgical outcomes of robot-assisted laparoscopic pancreaticoduodenectomy versus laparoscopic pancreaticoduodenectomy for periampullary neoplasms: A comparative study of a single center. Surg. Endosc..

[26] van Oosten AF (2020). Perioperative outcomes of robotic pancreaticoduodenectomy: A propensity-matched analysis to open and laparoscopic pancreaticoduodenectomy. J. Gastrointest. Surg..

[27] Kamarajah SK (2020). A systematic review and network meta-analysis of different surgical approaches for pancreaticoduodenectomy. HPB (Oxford).

[28] Vining CC (2020). Risk factors for complications in patients undergoing pancreaticoduodenectomy: A NSQIP analysis with propensity score matching. J. Surg. Oncol..

[29] Zhang T, Zhao ZM, Gao YX, Lau WY, Liu R (2019). The learning curve for a surgeon in robot-assisted laparoscopic pancreaticoduodenectomy: A retrospective study in a high-volume pancreatic center. Surg. Endosc..

[30] Shyr BU, Chen SC, Shyr YM, Wang SE (2018). Learning curves for robotic pancreatic surgery-from distal pancreatectomy to pancreaticoduodenectomy. Medicine.

