# Supplementary material for: Feasibility of simultaneous development of laparoscopic and robotic pancreaticoduodenectomy
Source: Sci Rep. 2023 Apr 16;13:6190. doi: 10.1038/s41598-023-33269-x (PMC10106461; doi:10.1038/s41598-023-33269-x)
Supplement: Supplementary file 1 — Supplementary Tables. [file 41598_2023_33269_MOESM1_ESM.docx]

**Supplementary Table 1. Patient characteristics between learning curve phases in LPD and RPD**

|  | LPD, n=39 | | | RPD, n=75 | | |
| --- | --- | --- | --- | --- | --- | --- |
|  | **PLC Phase** | **ALC Phase** | **p value** | **PLC Phase** | **ALC Phase** | **p value** |
| Patients, n | 13 | 26 |  | 18 | 57 |  |
| Sex (F/M) | 0/13 | 15/11 | < 0.001 | 12/6 | 30/27 | 0.42 |
| Age, year | 58.3 (54.9-69.6) | 72 (63-77) | 0.02 | 67.1 (55.3-76.9) | 65.5 (58.2-75.3) | 0.99 |
| BMI, kg/m^2^ | 23.6 (21.2-25.6) | 23.9 (21.1-25.6) | 0.94 | 24.4 (22.9-27.1) | 23.8 (22.3-26.9) | 0.42 |
| ASA score  I  II  III | 0  11 (85%)  2 (15%) | 0  13 (50%)  13 (50%) | 0.045 | 3 (17%)  8 (44%)  7 (39%) | 3 (5%)  31 (54%)  23 (40%) | 0.29 |
| Diagnosis  Ampullary cancer  Pancreatic ca  Cholangiocarcinoma  Duodenal cancer  Other malignancy  Benign disease | 8 (62%)  2 (15%)  0  0  2 (15%)  1 (8%) | 11 (42%)  2 (8%)  5 (19%)  1 (4%)  1 (4%)  6 (23%) | 0.33 | 5 (28%)  5 (28%)  4 (22%)  0  0  4 (22%) | 23 (40%)  8 (14%)  6 (11%)  0  4 (7%)  16 (28%) | 0.30 |
| Tumor size, cm | 2.5 (1.6-4.5) | 2.0 (1.0-3.2) | 0.31 | 2.1 (1.5-3.0) | 2.0 (1.0-2.9) | 0.40 |
| Soft pancreas | 12 (92%) | 24 (92%) | >0.99 | 17 (94%) | 54 (95%) | >0.99 |
| Dilated pancreatic duct | 6 (46%) | 9 (35%) | 0.51 | 4 (22%) | 14 (25%) | >0.99 |
| *BMI, body mass index; ASA,* *American Society of Anesthesiology*; *PLC, pre-learning curve; ALC, after-learning curve* | | | | | | |

| Supplementary Table 2. Patient characteristics between approaches in the PLC and ALC phase | | | | | | |
| --- | --- | --- | --- | --- | --- | --- |
|  | **PLC phase** | | | **ALC phase** | | |
|  | **LPD** | **RPD** | **p value** | **LPD** | **RPD** | **p value** |
| Patients, n | 13 | 18 |  | 26 | 57 |  |
| Sex (F/M) | 0/13 | 12/6 | <0.001 | 15/11 | 30/27 | 0.82 |
| Age, years | 58.3 (54.9-69.6) | 67.1 (55.3-76.9) | 0.28 | 72 (63-77) | 65.5 (58.2-75.3) | 0.07 |
| BMI, kg/m^2^ | 23.6 (21.2-25.6) | 24.4 (22.9-27.1) | 0.38 | 23.9 (21.1-25.6) | 23.8 (22.3-26.9) | 0.46 |
| ASA score  I  II  III | 0  11 (85%)  2 (15%) | 3 (17%)  8 (44%)  7 (39%) | 0.06 | 0  13 (50%)  13 (50%) | 3 (5%)  31 (54%)  23 (40%) | 0.43 |
| Diagnosis  Ampullary cancer  Pancreatic ca  Cholangiocarcinoma  Duodenal cancer  Other malignancy  Benign disease | 8 (62%)  2 (15%)  0  0  2 (15%)  1 (8%) | 5 (28%)  5 (28%)  4 (22%)  0  0  4 (22%) | 0.06 | 11 (42%)  2 (8%)  5 (19%)  1 (4%)  1 (4%)  6 (23%) | 23 (40%)  8 (14%)  6 (11%)  0  4 (7%)  16 (28%) | 0.75 |
| Tumor size, cm | 2.5 (1.6-4.5) | 2.1 (1.5-3.0) | 0.44 | 2.0 (1.0-3.2) | 2.0 (1.0-2.9) | 0.74 |
| Soft pancreas | 12 (92%) | 17 (94%) | >0.99 | 24 (92%) | 54 (95%) | 0.65 |
| Dilated pancreatic duct | 6 (46%) | 4 (22%) | 0.25 | 9 (35%) | 14 (25%) | 0.34 |
| *BMI, body mass index; ASA, American Society of Anesthesiology; PLC, pre-learning curve; ALC, after-learning curve* | | | | | | |
